# Therapeutic B cell depletion identifies immunoregulatory networks

**DOI:** 10.1172/JCI189442

**Published:** 2025-06-02

**Authors:** Carolina M. Polonio, Francisco J. Quintana

**Affiliations:** 1 Ann Romney Center for Neurologic Diseases, Brigham and Women’s Hospital, Harvard Medical School, Boston, Massachusetts, USA.; 2 Broad Institute of MIT and Harvard, Cambridge, Massachusetts, USA.; 3 Gene Lay Institute of Immunology and Inflammation, Brigham and Women’s Hospital, Massachusetts General Hospital, Harvard Medical School, Boston, Massachusetts, USA.

## Abstract

B cell depletion is a highly effective therapy in multiple sclerosis (MS), reducing inflammation and restoring immune balance. In this issue of the *JCI,* Wei et al. used single-cell RNA-Seq and flow cytometry, identifying the comprehensive effects of B cell depletion on the immune response, including an increase in antiinflammatory cerebrospinal fluid macrophages and elevated TNF-α expression by peripheral CD16^+^ monocytes. The authors also detected shifts in T cell populations that resulted in reduced myelin-reactive CD4^+^ T cells and the expansion of TIGIT^+^ Tregs. The findings uncover immunoregulatory mechanisms and suggest therapeutic strategies for MS and other autoimmune disorders.

## Immune dynamics via B cell depletion in multiple sclerosis

Multiple sclerosis (MS) is a chronic autoimmune disorder characterized by inflammatory demyelination and neurodegeneration within the CNS (1, 2). B cells play critical roles in MS pathogenesis through autoantibody production, antigen presentation, and cytokine-mediated immune activation (3, 4). B cell–depleting therapies, such as the humanized anti-CD20 monoclonal antibody ocrelizumab, revolutionized the treatment of MS by effectively reducing disease activity and slowing progression (5, 6). Indeed, the clinical success of B cell–targeting therapies highlights the importance of B cells for the control of T cell autoimmunity in MS. However, the broader effect on the immune response of CD20-targeting ocrelizumab remains unclear.

**Figure 1:**
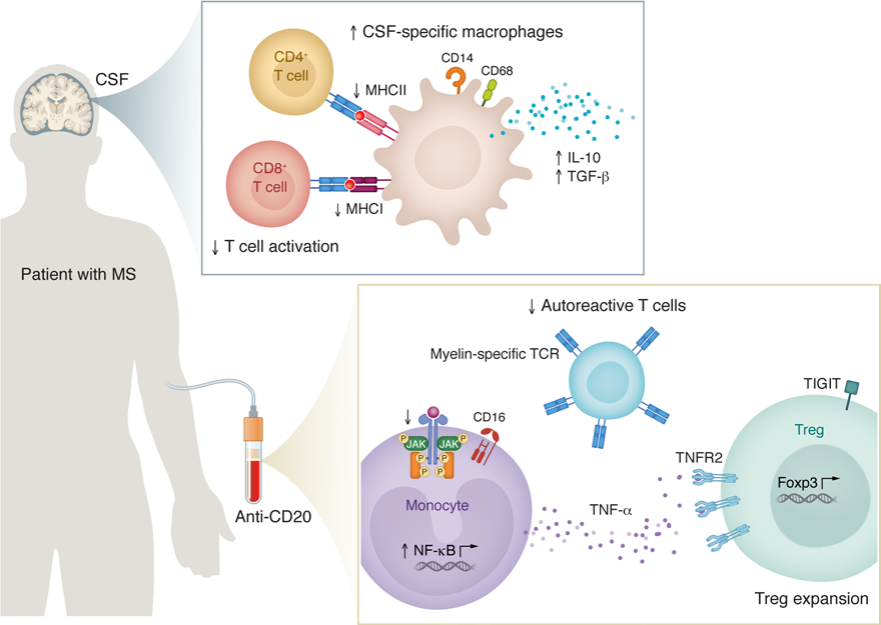
Immune reprogramming in patients with MS by B cell depletion therapy. B cell depletion increases CD14^+^CD68^+^ macrophages in the CSF of patients with MS. These macrophages exhibit reduced MHC class I and II expression, limiting T cell activation, while upregulating the antiinflammatory cytokines IL-10 and TGF-β, all of which promote an immunoregulatory environment. In the peripheral immune compartment, B cell depletion induces changes in monocytes, autoreactive T cells, and Tregs. CD16^+^ monocytes exhibit enhanced NF-κB signaling and increased TNF-α production, which support Treg expansion via TNFR2. In addition, B cell depletion reduces the frequency of myelin-reactive T cells, while increasing Treg abundance and function.

In this issue of the *JCI*, Wei et al. provide a comprehensive analysis of the broad immune effects of ocrelizumab in MS (7). Through elegant single-cell RNA-Seq (scRNA-Seq) and flow cytometric analyses of PBMCs and cerebrospinal fluid (CSF) samples, this study shines new light on the effects of CD20 targeting in MS, while identifying additional roles of B cells in immune regulation. Notable results include the detection of treatment-induced antiinflammatory CSF macrophages and elevated TNF-α expression in peripheral CD16^+^ monocytes, concomitant with a reduction in autoreactive CD4^+^ T cells and the expansion of TIGIT^+^Foxp3^+^ T cells (Figure 1). Collectively, these findings clarify the role of B cells and their therapeutic targeting in the regulation of immune system components.

## CNS-specific macrophages as a hallmark of immune restoration

In this study, Wei et al. analyzed CSF samples from patients with relapsing-remitting MS (RRMS) by scRNA-Seq before, and 6, 12, and 18 months after B cell depletion, alongside age-matched healthy controls (7). Strikingly, the authors detected a post-treatment increase in CD14^+^CD68^+^ macrophages in MS CSF samples, which brought their levels closer to those detected in healthy donors. DC clusters also showed modest post-treatment frequency increases, but no changes in antigen presentation or inflammatory transcriptional programs were detected.

During the investigation of the transcriptional response from CD14^+^CD68^+^ macrophages, a population of CSF-specific macrophages was identified. This subset, characterized by the expression of microglial and monocytic transcriptional modules, was expanded in patients following B cell depletion therapy. Notably, these macrophages exhibited increased expression of markers such as APOE and CSF1R, distinguishing them from other CNS macrophage and monocyte populations. Further analyses established that CSF-specific macrophages displayed decreased proinflammatory gene expression, concomitant with the upregulation of antiinflammatory molecules such as IL-10 and TGF-β. The reduced expression of genes linked to antigen presentation, including those encoding MHC class I and II, suggests a decreased potential for T cell activation (7).

Wei et al. identified distinct clusters within the CD14^+^CD68^+^ macrophage population (7). The CD14^+^CD68^+^ myeloid 1 (Mac 1) cluster showed the closest transcriptomic similarity to intermediate and nonclassical monocytes, whereas the Mac 2 cluster more closely aligned with classical monocytes. Collectively, these findings identify another role for B cells in the control of CSF antiinflammatory CD14^+^CD68^+^ macrophages, suggesting a link between peripheral and CNS immune reprogramming that contributes to therapeutic efficacy in MS.

## Peripheral immune reprogramming and the rise of regulatory monocytes

Wei et al. then investigated whether the changes induced in the CSF by B cell depletion were reflected in peripheral blood. The scRNA-Seq analysis of PBMC samples from patients with RRMS confirmed the peripheral deletion of B cells and the increase in CD16^+^ monocytes displaying transcriptional signatures of intermediate and nonclassical monocytes; flow cytometric analyses validated this post-treatment increase in circulating CD14^+^CD16^+^ monocytes. Differential gene expression analysis and flow cytometry detected increased TNF-α production and NF-κB signaling alongside the expression of regulatory markers, such as HIF1A and CD83. Gene set enrichment analysis (GSEA) further revealed enhanced TNF-α/NF-κB signaling and suppressed JAK/STAT pathway activity in CD16^+^ monocytes following B cell depletion. Interestingly, these transcriptomic changes were absent in CSF macrophages, emphasizing the tissue-specific immune modulation effects of B cell depletion (7).

These findings prompted the authors to investigate whether the post-treatment TNF/NF-κB upregulation was cell-type specific. Indeed, GSEA showed that TNF/NF-κB pathway activation occurred across most immune cell types after treatment, with CD16^+^ monocytes showing the highest pre- and post-treatment TNF-α expression, as validated at the protein level. In contrast, JAK/STAT pathway downregulation was restricted to CD16^+^ monocytes, while surviving or repopulating B cells displayed enhanced survival, metabolism, and JAK/STAT-related pathways. Interestingly, transcriptional analyses detected similarities in NF-κB pathway activation between B cells and myeloid cells, with distinct responses in T lymphocytes. These results revealed systemic yet lineage-specific immune reprogramming induced by B cell depletion therapy (7).

## T cell reprogramming shifts toward regulation and tolerance

Myelin-reactive T cells play a central role in MS pathology (8). Autoreactive CD4^+^ T cells, particularly Th1 and Th17 subsets, infiltrate the CNS, producing proinflammatory cytokines like IFN-γ, IL-17, and GM-CSF, which contribute to myelin damage and neuroinflammation (8). Wei et al. found that B cell depletion reduced effector memory CD4^+^ T cells (Tem) that expressed the peripheral helper T cell (Tph) markers (Tem-Tph) (PDCD1^lo^CXCR5^+^) in CSF and PBMCs. Central memory CD4^+^ T cells (Tcm) expressing T follicular helper (Tfh) cell (Tcm-Tfh) (PDCD^+^CXCR5^+^) markers were also decreased in the CSF. These T cell subset reductions coincided with an increase in blood naive T cells expressing SOX4 (SOX4^+^PECAM1^+^), suggesting their replenishment by recent thymic emigrants. Gene program activity analysis detected decreased Tfh and Th1 profiles across CSF and blood following ocrelizumab treatment, particularly within Tcm-Tfh, Tem-Tph, and intermediate Tregs (Treg Int) (7). Interestingly, Th17 cells were increased in the CSF Tem compartment but decreased in blood, suggesting a redistribution of CD4^+^ T cell subsets between the CNS and periphery.

To analyze autoreactive T cells, the authors used MHC class II (DRB1*15:01 or DRB1*04:01) tetramers loaded with the myelin epitopes myelin basic protein (MBP), proteolipid protein (PLP), and myelin oligodendrocyte glycoprotein (MOG) (7). They detected a decrease in myelin-reactive, double-positive (DRB1*15:01^+^ DRB1*04:01^+^) T cells in patients who had undergone B cell depletion. These decreases were accompanied by reduced frequencies of CD45RA^–^CXCR5^+^ Tfh and CCR6^+^CXCR3^–^ Th17 cells within myelin-specific T cell populations. These findings demonstrate that B cell depletion therapy reshapes the CD4^+^ T cell landscape by reducing pathogenic autoreactive subsets and modulating the expression of effector transcriptional programs.

## Pathways converge in myeloid and lymphoid cells

Deficits in Tregs have also been reported in MS (9). Following B cell depletion, Wei et al. identified a decrease in naive FOXP3^+^CCR7^+^ Tregs and intermediate FOXP3^+^FCRL3^+^ Tregs, alongside an increase in effector HLA-DR^+^CD74^+^ Tregs, suggesting a functional shift toward an effector phenotype (7). Ligand-receptor prediction analysis revealed potential interactions between TNF receptor 2 (TNFR2) on Tregs and TNF produced by myeloid cells, suggesting that monocyte-derived TNF may drive Treg expansion via TNFR2 signaling. Furthermore, B cell depletion increased TIGIT-expressing Tregs (10). Therefore, B cell depletion induces a functional shift in Tregs, characterized by increased effector and TIGIT-expressing Treg subsets, potentially driven by monocyte-produced TNF-α signaling via TNFR2.

## Immunoregulatory mechanisms and therapeutic opportunities for MS

In summary, the comprehensive analysis of the effects of B cell depletion on the immune system provided by Wei et al. establishes the reprogramming of myeloid and lymphoid cell responses in the CNS and the periphery (7). The shift toward antiinflammatory phenotypes in CSF macrophages, concomitant with the enrichment of regulatory monocytes and Tregs in the periphery, highlight the intricate crosstalk between immune cell types and tissue compartments. Furthermore, the reduction of autoreactive CD4^+^ T cells and the expansion of TIGIT-expressing effector Tregs underscore the therapeutic potential of immune modulation.

These findings advance our understanding of the role of B cells in MS pathogenesis, identifying unexpected effects of B cell depletion that might guide the identification of additional physiologic roles of B cells in immune regulation (7). Promising future directions of these studies include targeting CD16^+^ monocytes to induce immunomodulatory phenotypes while limiting pathogenic responses, using, for example, nanomaterial or engineered probiotics–based approaches (11, 12). Moreover, given the reported crosstalk of myeloid cells with cells of the nervous system (2), it will be important to determine the effect of these immunomodulatory monocytes with neurons and glial cells. In summary, the study by Wei et al. suggests basic immunoregulatory mechanisms as well as potential therapeutic interventions for MS and other autoimmune diseases (7).

## Version 1 06/02/2025

Electronic publication

## References

[1] Reich DS (2018). Multiple Sclerosis. N Engl J Med.

[2] Charabati M (2023). Multiple sclerosis: Neuroimmune crosstalk and therapeutic targeting. Cell.

[3] Comi G (2021). Role of B Cells in Multiple Sclerosis and Related Disorders. Ann Neurol.

[4] Cao Y (2015). Functional inflammatory profiles distinguish myelin-reactive T cells from patients with multiple sclerosis. Sci Transl Med.

[5] Hauser SL (2017). Ocrelizumab versus Interferon Beta-1a in Relapsing Multiple Sclerosis. N Engl J Med.

[6] Cencioni MT (2021). B cells in multiple sclerosis - from targeted depletion to immune reconstitution therapies. Nat Rev Neurol.

[7] Wei J (2025). Transcriptomic profiling after B cell depletion reveals central and peripheral immune cell changes in multiple sclerosis. J Clin Invest.

[8] Sospedra M, Martin R (2005). Immunology of multiple sclerosis. Annu Rev Immunol.

[9] Wagner CA (2019). Pathogenic T cell cytokines in multiple sclerosis. J Exp Med.

[10] Joller N (2014). Treg cells expressing the coinhibitory molecule TIGIT selectively inhibit proinflammatory Th1 and Th17 cell responses. Immunity.

[11] Kenison JE (2020). Tolerogenic nanoparticles suppress central nervous system inflammation. Proc Natl Acad Sci U S A.

[12] Sanmarco LM (2023). Lactate limits CNS autoimmunity by stabilizing HIF-1α in dendritic cells. Nature.

